# Simple and Robust Realtime QRS Detection Algorithm Based on Spatiotemporal Characteristic of the QRS Complex

**DOI:** 10.1371/journal.pone.0150144

**Published:** 2016-03-04

**Authors:** Jinkwon Kim, Hangsik Shin

**Affiliations:** 1 Advanced Safety Vehicle Development Team, Hyundai Motors, Hwaseong-si, Gyeonggi-do, Republic of Korea; 2 Department of Biomedical Engineering, Chonnam National University, Yeosu, Jeollanam-do, Republic of Korea; University at Buffalo, UNITED STATES

## Abstract

The purpose of this research is to develop an intuitive and robust realtime QRS detection algorithm based on the physiological characteristics of the electrocardiogram waveform. The proposed algorithm finds the QRS complex based on the dual criteria of the amplitude and duration of QRS complex. It consists of simple operations, such as a finite impulse response filter, differentiation or thresholding without complex and computational operations like a wavelet transformation. The QRS detection performance is evaluated by using both an MIT-BIH arrhythmia database and an AHA ECG database (a total of 435,700 beats). The sensitivity (SE) and positive predictivity value (PPV) were 99.85% and 99.86%, respectively. According to the database, the SE and PPV were 99.90% and 99.91% in the MIT-BIH database and 99.84% and 99.84% in the AHA database, respectively. The result of the noisy environment test using record 119 from the MIT-BIH database indicated that the proposed method was scarcely affected by noise above 5 dB SNR (SE = 100%, PPV > 98%) without the need for an additional de-noising or back searching process.

## Introduction

An electrocardiograph (ECG) is a graphical representation of the electrical activity of a heart over time, and it is the most basic examination method that can be used for cases of heart disease. However, it may be necessary to record and analyze long-term ECGs since the symptoms of some types of heart disease are generally intermittent. Thus, research related to automatic ECG processing algorithms has been actively conducted over the last several decades. The first step of an automatic ECG processing algorithm involves detecting the QRS complex. With the results of the QRS complex detection, other fiducial points in the ECG, such as P, Q, S, or T, can also be detected to provide further information [1–4]. As a result, QRS complex detection methods have a considerable influence on the performance levels in subsequent steps of the algorithm. Nowadays, many ECG-based devices have been developed for use toward personalized healthcare, and the industry is evolving to provide ubiquitous and mobile healthcare. Since automatic ECG processing algorithms have been developed for use with every kind of ECG-related application, the need for stable, noise-robust signal processing techniques has been increasing to provide ubiquitous use. Therefore, current research on QRS complex detection algorithms has focused on increasing the performance in terms of the detection accuracy, computational burden, and noise-robustness of the detection [5–7].

The accuracies of modern QRS complex detection algorithms are already quite high. Though many QRS complex detection algorithms show over 99% in sensitivity and a positive predictive value [4, 8–16], most only used the MIT-BIH arrhythmia database [17] or AHA ECG database [18] during their evaluation. Moreover, most of them are offline algorithms, which cannot be used in causal systems. Research related to the Pan & Tompkins QRS detection algorithm [14], the most commonly used algorithm, led to the development of a real-time QRS complex detection algorithm that operates according to the slope, amplitude, and width. The results showed the sensitivity (SE) of this algorithm was of 99.75% with a positive predictive value (PPV) of 99.54%. Lee *et al*. [12] used a modified spatial velocity method to measure the energy of the QRS complexes for reliable detection. The algorithm was also evaluated using the MIT-BIH arrhythmia database and showed SE results of 99.88% and a PPV result of 99.69%. Afonso *et al*. [9] detected QRS complexes based on the sub-band components of an ECG by using a filter bank, achieving an SE result of 99.59% and a PPV result of 99.56% with the same database.

To maintain a high level of accuracy, even in noisy environments, Christov proposed an adaptive threshold method that combines three parameters: an adaptive slew-rate value, another parameter to reduce the effect of the high-frequency noise, and a third parameter to detect low-amplitude beats [11]. This method achieved an SE result of 99.69% and a PPV result of 99.80%. Yeh *et al*. [16] proposed offline QRS complex detection based on a difference operation method to produce more reliable and faster detection involving a relatively simple operation. The algorithm had a 99.89% SE and a 99.95% PPV.

The current trend in research could be summarized to develop a highly accurate algorithm that can maintain a high level of accuracy, even in very noisy environments, in spite of having a high computational burden. For this, we have designed a new QRS complex detection method that takes into account the characteristics of the ECG signal. To represent the electric activity of the ventricle, the waveform for the QRS complex has a regular range in terms of the amplitude and variation in speed. The proposed QRS complex detection method consists of two detection criteria. Since the electrical activities caused by the ventricular beats have a regular range for the energy level within a certain frequency band, the first criterion evaluates whether or not the energy in a specific frequency band exceeds a certain level. The second criterion involves checking whether or not the energy appears or disappears after a certain period (approximately the duration of the QRS complex) since the heart, the energy source, has a quiet interval between heartbeats. In addition, the algorithm operates in a stable manner by using additional parameters in various environments, such as with surrounding noise or other noises that change in amplitude. To evaluate the robustness of the proposed algorithm, data from multiple databases were processed, including the MIT-BIH arrhythmia database and the AHA ECG database. Moreover, various aspects of the proposed algorithm were inspected, and the noise analysis, including the effects of the noise and the signal amplitude, were also included.

## Materials and Methods

**Fig 1:**
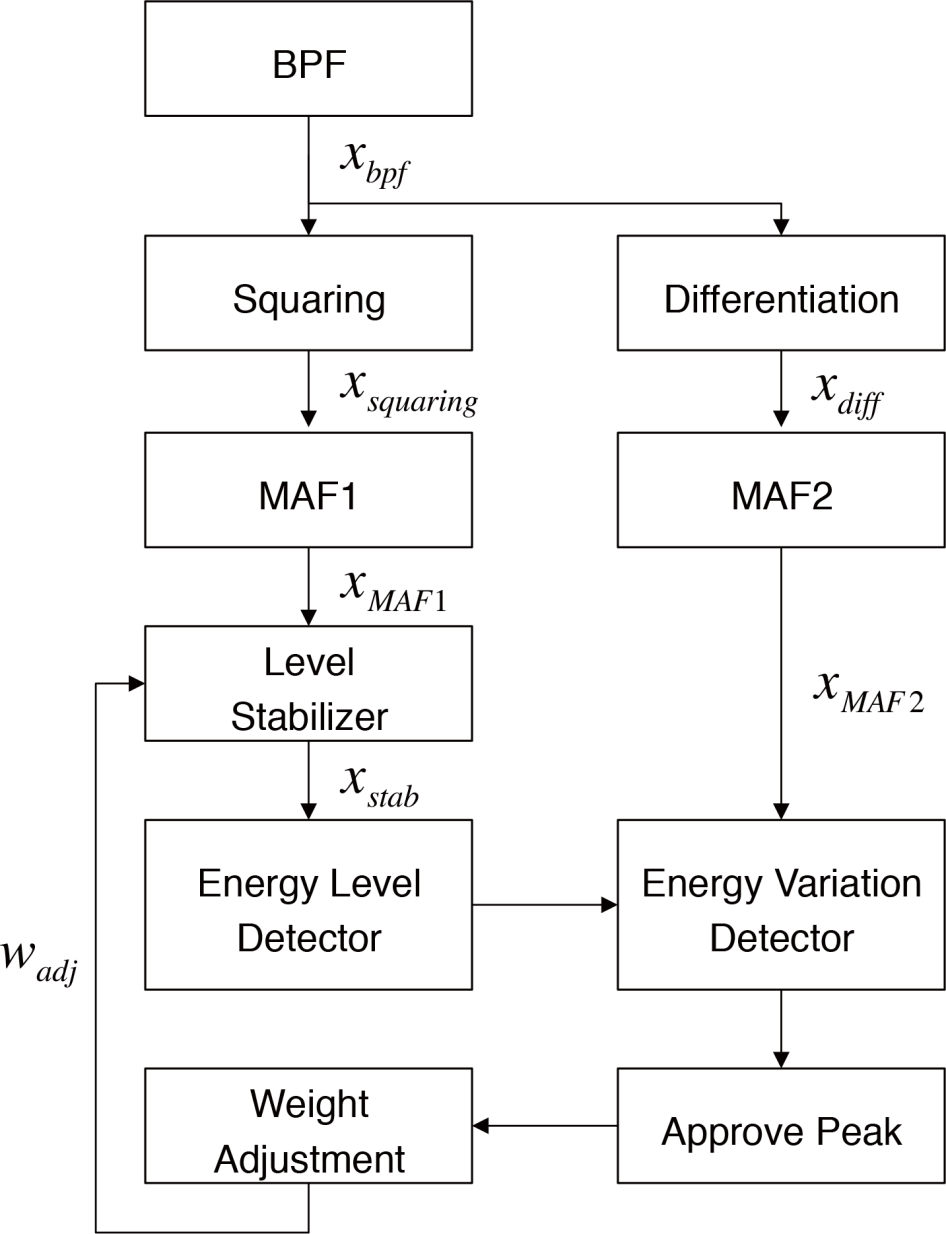
Procedure for the proposed QRS detection algorithm.

The proposed algorithm has the advantages of providing a stable performance, of using intuitive criteria based on the characteristics of the QRS complex, and requiring simple computation. The algorithm detects the QRS complex by examining two features. The first feature is the level of energy of the waveform and the second is the variation within a given time duration. These features reflect the characteristics of the QRS complex, which means that the major energy components are within a certain frequency band (mainly 5 to 25Hz) and that there is a variation in energy components after a certain time duration (the duration of the QRS complex). We detected the QRS complex using these features because, unlike the QRS complex, noise components generally occur in different frequency bands or are present continuously. Fig 1 shows a block diagram of the proposed QRS complex detection method. Before the assessment of the above features, the input ECG signal passes through the band pass filter (BPF). The BPF is a 64-tap finite impulse response (FIR) filter, and it has a pass band from 5 to 25 Hz, where the QRS complexes have their main energy components.

### Energy Level Detection

To calculate the energy level of the signal power components of the filtered signal, *x*_*squared*_ was obtained by squaring. Then, the moving average filter (MAF1) was applied to the squared signal in order to acquire the average power (*x*_*MAF*1_) within a certain time range. Then, peak detection was performed using adaptive thresholds based on the output signal of the MAF1. The above process is designed to measure the amplitude of the energy within a certain time range.

A detailed configuration of the control parameter and its meaning was obtained in the following manner. MAF1 is used to calculate the energy in a particular frequency range. Thus, the length of MAF1 should be similar to the duration of the QRS complex. In general, the duration of the QRS complex is of no more than 0.1 seconds in a normal state, but it can increase to 0.15 seconds in an abnormal state. Therefore, the length of MAF1 needs to be longer than 0.15 seconds to cover the QRS complex. However, the length of MAF1 should have the shortest length as possible because the filtering result of the QRS complex could be influenced by adjacent features such as P or T waves of the ECG waveform when too wide MAF1 is used. Therefore, in this study, the length of MAF1 was set to 0.15 seconds. Eq (1) shows the formula for MAF1, where *N*_1_ is the number of samples within 0.15 seconds.

$$x_{MAF1}[n]=\frac{1}{N_{1}}\sum_{k=<N_{1}>}x_{squared}[k]$$ (1)

An adaptive threshold was applied to the output signal of MAF1 to explore the peak. The adaptive threshold is composed of two kinds of thresholds: a signal threshold (*th*_*sig*_) and a noise threshold (*th*_*noise*_). During adaptive threshold detection, the signal threshold represented in Eq (2) comes along the signal when the threshold is lower than the value of the waveform. Otherwise, the signal threshold is controlled by both the previous signal and the threshold level for the noise. In this study, the decay rate (*r*_*decay*_) is set to decrease the signal threshold with a specified rate defined through a combination of the fixed decay constant and the peak-noise amplitude ratio. The decay rate is basically designed to produce a decrease from the initial value of the signal threshold of 5.75% per second. This rate is adapted by considering the refractory period (RP) as 150 ms and a general human beat-to-beat interval of 700 ms (Eq (3)). The decay rate is finally determined by multiplying the noise-peak amplitude ratio. The noise-peak amplitude ratio reflects the noise level to the signal peak amplitude ratio, and it changes the decay rate with the application rate, *r*_*d*_. The noise threshold also contributes to the change in the signal threshold with an application rate *r*_*n*_. The application rate indicates the degree of the contribution of each input parameter to the value obtained as the result. The signal threshold, *r*_*d*_ and *r*_*n*_, are determined to be 5% and 3%, respectively, and were empirically determined. For other conditions, such with a refractory period or *r*_*nr*_ times the noise threshold exceeding the signal threshold, the signal threshold will be set to the previous value or to *r*_*nr*_ times of noise threshold. Empirically, *r*_*nr*_ was set as 1.75.

$$th_{sig}=\{\begin{array}{ll} \;x_{MAF1}[n] & x_{MAF1}[n]>th_{sig}[n]\\ \;th_{sig}[n-1] & \text{in refractory period}\\ \;r_{nr}th_{noise}[n-1] & r_{nr}th_{noise}[n-1]>th_{sig}[n-1]\\ \;r_{decay}th_{sig}[n-1]+r_{n}th_{noise}[n-1] & otherwise \end{array}$$ (2)

$$r_{decay}=\left(1-\frac{1}{(0.5-RP)f_{s}}\right)\left(1-r_{d}\frac{th_{noise}[n-1]}{V_{prepeak}}\right)$$ (3)

The noise threshold, shown in Eq (4), depends on the previous noise threshold level and on the filtered signal level. It is used to determine the next noise level in the signal. In Eq (4), the application rate, *r*_*s*_, is set to 0.1%. Finally, for the energy level detection, the QRS candidate is determined at the end of the accompanying signal and threshold if the candidate is not within the refractory period.

$$th_{noise}[n]=\{\begin{array}{ll} \;x_{MAF1}[n] & th_{noise}[n]>x_{MAF1}[n]\\ \;th_{noise}[n-1]+r_{s}x_{MAF1}[n-1] & otherwise \end{array}$$ (4)

### Energy Variation Detection

The second criterion checked whether the energy variation has a proper value within a certain time interval. The energy variation means an increase and decrease in the energy generated from the QRS complex. Though the amplitude of the ECG could be lower than 0.39 mV in the low ECG case [19], the amplitude of a normal QRS complex has been known to be greater than 0.5 mV in general. Moreover, as mentioned above, the normal QRS duration is of 0.06 to 0.1 seconds. These characteristics can be used to define the rule for the speed of the rising and falling of the QRS complex, which could be from 3.9 mV/s to 35 mV/s or greater. Although there is a huge difference within this range, we could set the boundary of the QRS energy variation.

The rising and falling speed of the energy is evaluated by obtaining the difference signal of the squared signal (*x*_*diff*_) and then applying MAF2 (Eq (5)). MAF2 was used to calculate the change over a certain period of time. Thus, the length of MAF2 (*N*_2_) should be similar to the duration of the QRS complex, and moreover, it should be long enough to cover the QRS duration that could occur a representative human. Therefore, in this study, MAF2 was set to a length of 0.2 seconds. To evaluate the energy variation criterion, the output signal of MAF2 was assessed to ensure that that it has the proper range where the peak was detected in the energy level detection stage.

$$x_{MAF2}[n]=\frac{1}{N_{2}}\sum_{k=<N_{2}>}x_{diff}[k]$$ (5)

Three criteria were designed and used to judge the variation in energy. The first criterion involved the minimum requirement for the QRS energy variation. We set the maximum absolute energy variation speeds to exceed ±0.45 mV per 0.2 seconds within 0.2 seconds earlier or later than the peak of the MAF1. The second criterion was that the magnitude of the changes during the 0.4 seconds should be greater than 2 mV per 0.4 seconds. The last criterion is that the maximum absolute energy variation speeds should not exceed ±20 mV/s within 0.2 seconds before or after the MAF1 peak. Every criterion was commonly adjusted to 5% of the value, considering the averaging effect of MAF2. If they exceed the criterion, they are regarded as noise. The QRS complex is determined only when all of the above conditions have been satisfied.

### Energy Level Stabilizer

The QRS complexes were detected according to the results obtained from these two detection stages: the energy level detection and the energy variation detection. However, the amplitude of the input ECG signal could affect the energy level detection because it relies on the amplitude thresholds. Therefore, a level stabilizer that generates the stabilized signal (*x*_*stab*_) could relieve the abrupt changes in the signal level and improve the performance of the adaptive threshold peak detection method by regulating the signal level over a specific range. The weights of the level stabilizer were adjusted according to the amplitude of the detected peak. The signal was applied to adaptive thresholds reduce the effects of the variation in the amplitude of the ECG. To this end, a weight adjustment block was added as a regulator. This block controls the weights of the level stabilizer, *w*_*stab*_, to produce a stable signal level for the MAF1 output (Eq (6)).

$$x_{stab}[n]=\frac{w_{stab}[n]}{N_{1}}\sum_{k=<N_{1}>}x_{squared}[k]$$ (6)

To control the weights, the weight in every cycle is adjusted with *w*_*adj*_ when it was greater or less than a predefined reference value (*level*_*stab*_) of 0.5 mV (Eq (7)). The amount of weight adjustment, *w*_*adj*_, is determined with Eq (7), where *V*_5*peak*_, *r*_*a*_ and *r*_*b*_ are the 5 recent peaks and an application rate of 10% and 5%, respectively. Through this process, the QRS complex was reliably detected, even when the amplitude of the signal had varied.

$$\begin{array}{l} w_{\mathrm{stab}}[n+1]=w_{\mathrm{stab}}[n]+w_{\mathrm{adj}}[n]\\ w_{\mathrm{adj}}[n]=\left\{ \;\begin{array}{l} \text{arg min}\left(r_{a}\left({\mathrm{level}}_{\mathrm{stab}}-\mathrm{median}(V_{5\;\mathrm{peak}})\right),\;r_{b}{\mathrm{level}}_{\mathrm{stab}}\right),\;{\mathrm{level}}_{\mathrm{stab}}>\mathrm{mean}(V_{5\;\mathrm{peak}})\\ \text{arg min}\left(r_{a}\left({\mathrm{level}}_{\mathrm{stab}}-\mathrm{median}(V_{5\;\mathrm{peak}})\right),\;-r_{b}{\mathrm{level}}_{\mathrm{stab}}\right),\;\text{otherwise} \end{array}\right. \end{array}$$ (7)

**Fig 2:**
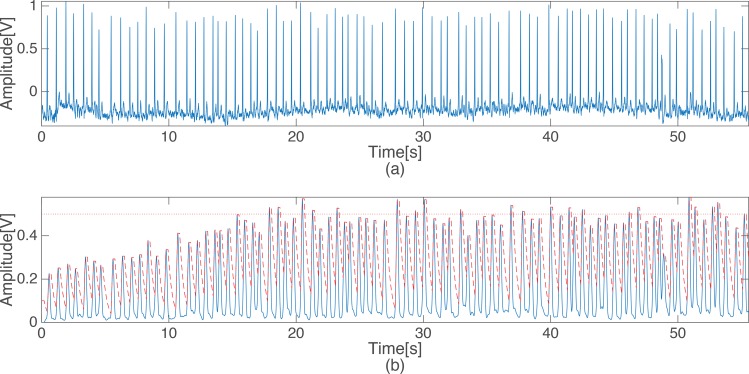
The original ECG signal and the scaled output of the level stabilizer (the dashed line is the energy level threshold.)

Fig 2 shows the compensation effects on the amplitude of the ECG. In Fig 2(B), the signal that was applied to the thresholds does not reach the predefined average value of 0.5 mV for MAF1 from the beginning. However, we could confirm that the signal level gradually increases and becomes similar to the *level*_*stab*_. The parameter values used for the above criterion were set to detect the QRS complexes in a stable manner when the signal was around a predefined average value. Throughout this process, the proposed algorithm reliably detects QRS complexes at various amplitudes of the input signal.

## Results and Discussion

### Database

In this study, the performance of the proposed QRS detection algorithm was evaluated by analyzing arrhythmia signals acquired from a variety of subjects. The evaluation was based on 183 records (435,801 beats) comprised of 48 records from the MIT-BIH arrhythmia database and 135 records from AHA ECG database (1000–7000 series).

### Statistical Methods

The proposed QRS complex detection algorithm was evaluated in terms of its sensitivity and positive predictive value, and the corresponding equations are shown below where the TP, FN, and FP refer to a true positive, a false negative, and a false positive result, respectively.

$$\text{Sensitivity}(\text{SE})=\frac{TP}{TP+FN}\times 100(\%)$$ (8)

$$\text{Positive Predictivity Value}(\text{PPV})=\frac{TP}{TP+FP}\times 100(\%)$$ (9)

### Evaluation 1: MIT-BIH and AHA Databases

**Fig 3:**
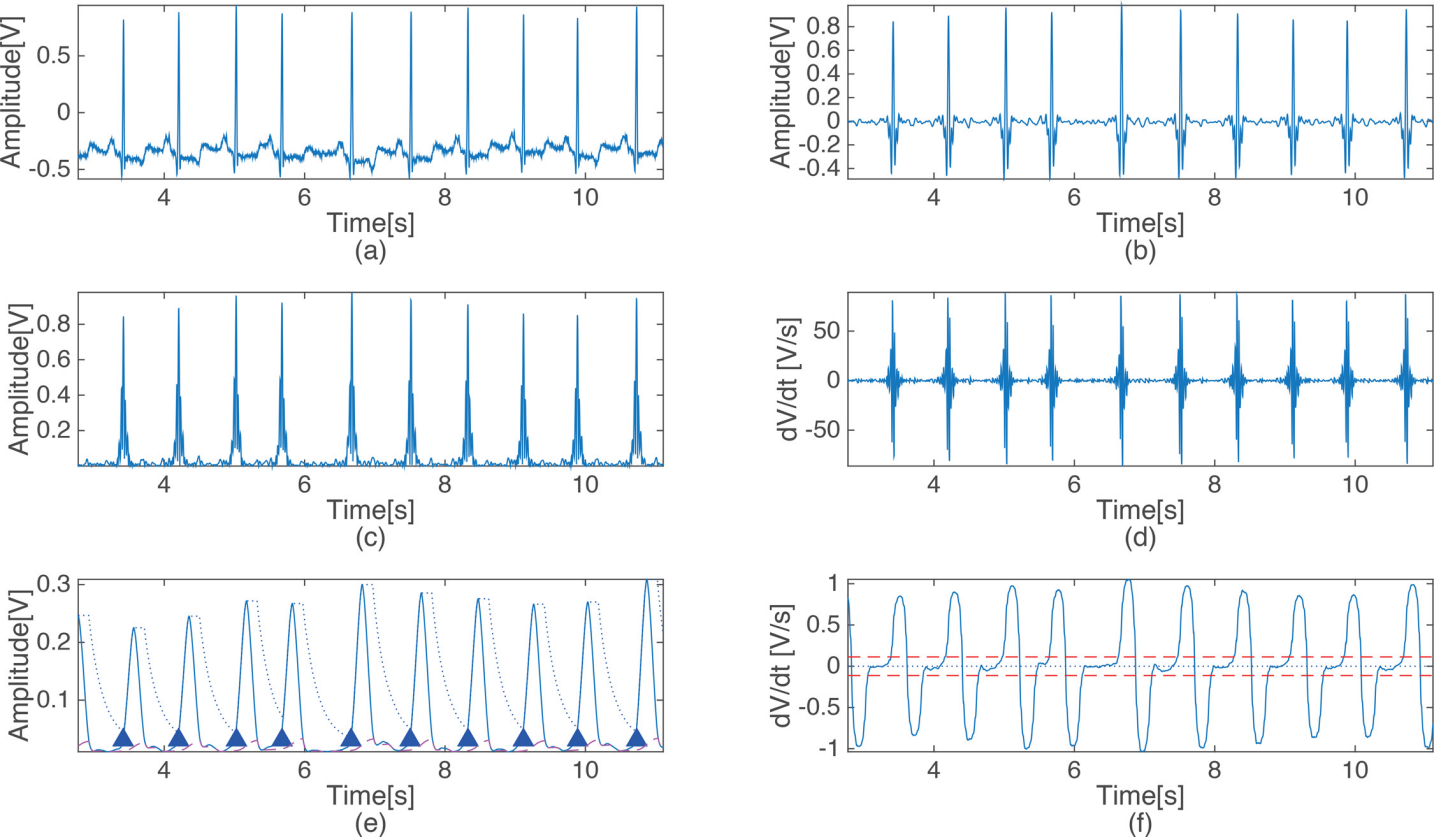
Output signal of each processing stage of the proposed algorithm. (a) Original ECG signal, (b) Band pass filtered ECG signal, (c) Output signal of the squaring, (d) Output signal of the difference block, (e) Output signal and threshold after MAF1 & level stabilization, (f) output signal and threshold of MAF2.

Fig 3 shows the steps of the proposed detection method. Each waveform in Fig 3 represents the output signal of each of the stage of the proposed detection method, as presented in Fig 1. Fig 3(A) shows the ECG of record 100 of the MIT-BIH arrhythmia database, and Fig 3(B), 3(C), 3(D), 3(E) and 3(F) show the output signals of the BPF, squaring, differentiation, level stabilizer and MAF2, respectively. The dotted line and the dashed in Fig 3(E) indicate the signal threshold and the noise threshold, respectively. The triangles indicate where the QRS complexes occur, and the dotted line in Fig 3(F) indicates 0 to verify the zero crossing while the dashed lines represent the first criterion of the energy variation detection.

S1 Table provides a summary of the results of the proposed algorithm based on the MIT-BIH arrhythmia database and the results of other algorithms evaluated using the same database. The proposed QRS complex detection method showed a very good overall detection performance for the MIT-BIH arrhythmia database. The average performance was of 99.90% SE and 99.91% PPV. These results are definitely superior to those found in earlier studies [4, 8–12, 14, 15] but are similar to those obtained in others [13, 16]. For comparison, S1 Table represents the most widely used algorithm [14], the highest performance reported to date [13], and a recently developed algorithm [8].

An evaluation of the results using the AHA ECG database indicated that the proposed algorithm was the best among any presented in previously published studies [14, 15, 20–23], as shown in Table 1. Statistically, in an AHA ECG database, the proposed QRS detection method shows an SE of 99.84% and a PPV of 99.84%. The above results based on the MIT-BIH and the AHA ECG databases indicate that the proposed QRS complex detection algorithm had a good overall detection performance. Overall, the proposed algorithm shows an SE of 99.85% of SE and a PPV of 99.86% for 435,700 beats.

**Table 1:** The results of the evaluation of the proposed algorithm and others based on the AHA ECG database

File number	TP	FP	FN	SE	PPV
**Proposed Algorithm**	325679	519	557	99.84	99.84
**Serafim Tabakov, *et*. *al*.** [15]	322328	2205	1085	99.32	99.66
**Ivan A Dotsinsky, *et*. *a*l.** [20]	324739	1376	1411	99.57	99.58
**U. Kunzmann, *et*. *al*.** [21]	181564	4446	309	97.61	99.83
**J. Pan, *et*. *al*.** [14, 23]*	64404	4166	8381	88.49	93.92
**H. So, *et*. *al*.** [22, 23]*	69197	623	3588	95.07	99.11

*Only 30 records (1201~1210, 2201~2210, 3201~3210) of AHA ECG database were evaluated.

**Fig 4:**
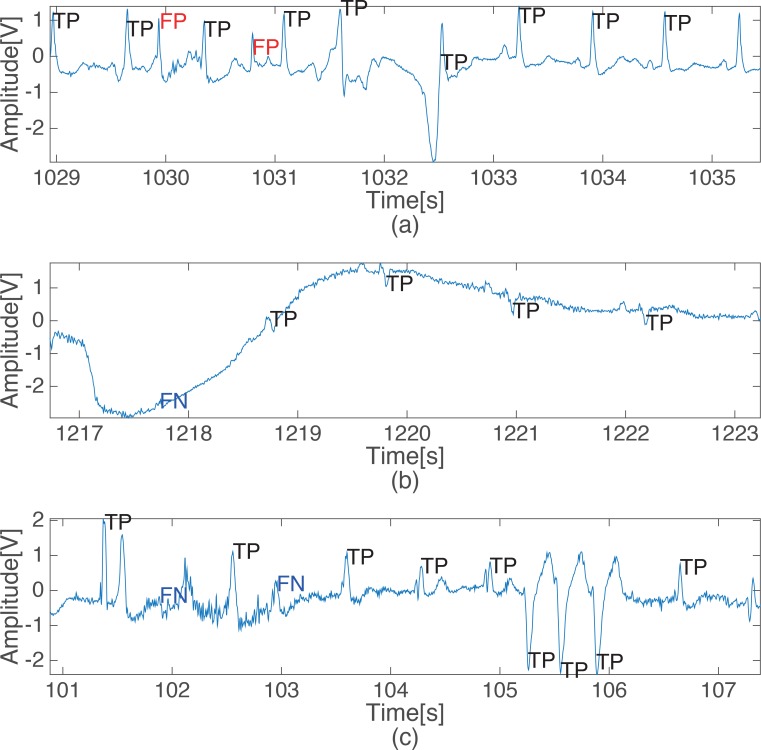
Examples of incorrect detection from some subjects who have over 20 FPs or FNs. (a) False positive detection in record 105, (b) False negative detection in record 108, (c) False negative detection in record 203.

The characteristics of the erroneous detections performed by the proposed QRS detection algorithm are analyzed by showing representative examples of incorrectly detected beats in Fig 4, which features records 105, 108 and 203 from MIT-BIH database with 20 or more beats that were incorrectly detected. Record 105 has extensive severe peak noise, as shown in Fig 4(A), and this created many FPs. However, record 108 includes a severe baseline movement or saturation, which leads to a considerable number of false negatives, as shown in Fig 4(B). Moreover, record 203 includes many irregular beats in the amplitude and shape, which produces many incorrect detections. However, it is challenging even for physicians to detect QRS complexes correctly without other lead signals in the three records mentioned above due to the severe noise or saturation regions that exist in those records. As shown in S1 Table, all of the other algorithms that were tested also returned many incorrect detection results from these same records.

### Evaluation 2: Evaluation in a Noisy Environment

**Fig 5:**
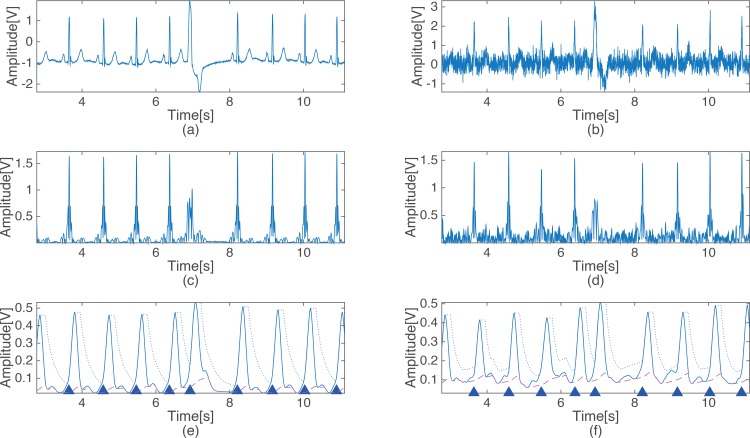
Output signals of the proposed algorithm with a clean ECG signal and a contaminated ECG signal. (a) clean ECG signal, (b) contaminated ECG signal (SNR = 6 dB).

The proposed QRS complex detection algorithm was evaluated in a noisy environment to test the robustness in the presence of random noise. The proposed algorithm adopts an adaptive threshold for the noise component in order to maintain accuracy in a noisy environment. Fig 5(A) shows the output signal of each of the criteria block of the proposed algorithm under normal circumstances with a low value for the noise power. The dashed lines in Fig 5(A) and 5(B) are the noise thresholds, and the dotted lines represent the signal threshold. The noise threshold in Fig 5(B) increased slightly relative to that in Fig 5(B) due to the increase in the noise power (SNR = 6 dB). Since the noise threshold examines the output signal passing through the BPF, raising the noise threshold does not appear prominently, even with noise. Through this process, the proposed algorithm prevents false detection in a noisy environment.

Previous research [10] analyzed the decline in the performance as a result of variation in the SNR in order to evaluate the robustness of the algorithm. In this study, the same conditions were used to conduct an evaluation, and the results were compared. The detection performance was evaluated using record 119 of the MIT-BIH arrhythmia database with an SNR that varied from 0 dB to 30 dB. Table 2 shows these results and also shows that the proposed algorithm has a higher noise stability when compared to that of a recent study [10]. In particular, even in the absence of an additional de-noising process, the proposed QRS complex detection method was scarcely affected by noise above a 5 dB SNR (SE = 100%, PPV > 98%).

**Table 2:** The evaluation of the proposed algorithm and others under a variety of noisy environments.

SNR (dB)	Proposed Algorithm					Szi-Wen Chen, *et*. *al*. [10]			
						with wavelet denoising		without wavelet denoising	
	TP	FP	FN	SE	PPV	SE	PPV	SE	PPV
0	1980	115	7	99.80	94.61	93.85	92.82	0.28	100
1	1986	104	1	99.95	94.75	97.21	95.87	0.28	100
2	1987	81	0	100	95.53	97.77	99.15	0.28	100
3	1987	68	0	100	96.46	99.16	99.16	0.28	100
4	1987	40	0	100	97.59	99.72	99.17	0.28	100
5	1987	31	0	100	98.08	100	98.9	0.28	100
6	1987	24	0	100	98.81	100	99.44	0.28	100
7	1987	13	0	100	99.35	100	99.44	0.56	100
8	1987	6	0	100	99.75	100	99.72	1.4	100
9	1987	2	0	100	99.85	100	99.72	2.79	100
10	1987	1	0	100	100	100	99.72	8.66	93.94
11	1987	0	0	100	100	100	99.72	17.32	92.54
12	1987	1	0	100	100	100	99.72	29.33	89.74
13	1987	0	0	100	100	100	99.72	41.06	83.52
14	1987	0	0	100	100	100	99.72	53.63	78.05
15	1987	0	0	100	100	100	100	65.08	71.91
16	1987	0	0	100	100	100	100	72.63	65.16
17	1987	0	0	100	100	100	100	81.28	59.51
18	1987	0	0	100	100	100	100	91.06	56.5
19	1987	0	0	100	100	100	100	96.65	56.81
20	1987	0	0	100	100	100	100	100	91.33
21	1987	0	0	100	100	100	100	100	98.62
22	1987	0	0	100	100	100	100	100	100
23	1987	0	0	100	100	100	100	100	100
24	1987	0	0	100	100	100	100	100	100
25	1987	0	0	100	100	100	100	100	100
26	1987	0	0	100	100	100	100	100	100
27	1987	0	0	100	100	100	100	100	100
28	1987	0	0	100	100	100	100	100	100
29	1987	0	0	100	100	100	100	100	100
30	1987	0	0	100	100	100	100	100	100

## Conclusion

In this study, we have designed a new QRS complex detection method that takes into account the characteristics of the ECG signal. The proposed QRS complex detection method is based on two detection criteria that have a low computational burden. In addition, the algorithm operates in a stable manner by using additional parameters that correspond to various environments, such as when there is surrounding noise or changes in the signal amplitude. The proposed algorithm was evaluated by using the MIT-BIH arrhythmia and the AHA ECG databases. It showed a very high detection performance for both databases. Specifically, the detection results based on the MIT-BIH arrhythmia database indicated that the proposed QRS complex detection method produced clearly erroneous results only when the detection was carried out in a severely noisy or saturated region. Moreover, the performance of the proposed algorithm was maintained at up to 5 dB SNR, as is shown in the evaluation where the SNR varied. One of the factors that contributes to the improvement in the performance is the level stabilization technique. We showed that the QRS detection methods based on the thresholds work properly when the amplitude of the input signal is maintained, even when the thresholds are adaptively applied. However, the amplitude of the ECG signal can change considerably depending on the person who is measured, the measuring equipment, and the sensor positions. To account for these changes, the proposed algorithm stabilizes the amplitude of the signal to which the thresholds are applied by adjusting the coefficients of the MAFs.

Consequently, the proposed algorithm shows adequate or even outstanding performance even though it only depends on basic signal processing procedures, such as FIR or MA filtering. In other words, the proposed algorithm has a suitable performance without high computational operations, such as a wavelet, and it may dramatically reduce the computational load. The computational complexity of the decomposition and reconstruction process of wavelet is known as *O*(*N*) in both cases where *N* is a number of time samples. However, computational complexity of algorithms could be increased in wavelet-based QRS detection because additional processing steps are generally needed after wavelet processing. The computational complexity of the whole procedure of proposed QRS detection algorithm is *O*(*N*) because it is a combination of linear systems based on FIR filtering that consists *N* times of multiplication and *N*-1 times of addition. It is the same level with the existing low complexity algorithm proposed by Yeh *et al*., which was proved the low computational load compared with wavelet-based method [16].

Considering that the expansion of the ECG-related device and the application toward personalized healthcare, a low computational load and ease of implementation are quite important environments with limited resources, such as when using mobile or consumer devices. Moreover, a simple, robust realtime algorithm is important to maintain a suitable performance during ubiquitous use because the computational load could increase in a noisy environment. From this point of view, the proposed algorithm, which only depends on the most common types of preprocessing methods such as FIR BPF and MAFs and a widely used thresholding technique [10, 11, 15], has the advantage in that it is simple, and moreover it shows superior performance compared with other realtime algorithms. It is our belief that the robust performance and low computational burden make this detection method useful for application in a limited environment, such as for *u*-healthcare with mobile or home healthcare devices. The source code used in this research is also available from website: https://github.com/HangsikShin/QRS-Detection/tree/master/Simple-and-Robust-Realtime-QRS-Detection-Algorithm-based-on-Spatiotemporal-Characteristic-of-the-QRS-Complex.

## Supporting Information

S1 Table The results of the evaluation of the proposed algorithm and others based on MIT-BIH arrhythmia database. (DOCX)
