# Supplementary material for: Simple and Robust Realtime QRS Detection Algorithm Based on Spatiotemporal Characteristic of the QRS Complex
Source: PLoS One. 2016 Mar 4;11(3):e0150144. doi: 10.1371/journal.pone.0150144 (PMC4778940; doi:10.1371/journal.pone.0150144)
Supplement: S1 Table — (DOCX) [file pone.0150144.s001.docx]

**Supporting Information**

**S1 Table. The results of the evaluation of the proposed algorithm and others based on MIT-BIH arrhythmia database**

| Subject | Total | Proposed Algorithm | | | | | Jianpu Pan, et. al. [14] | | | | | Cuiwei Li, et. al. [13] | | | | | Mourad Adnane, et. al. [8] | | | | |
| --- | --- | --- | --- | --- | --- | --- | --- | --- | --- | --- | --- | --- | --- | --- | --- | --- | --- | --- | --- | --- | --- |
|  |  | TP | FP | FN | SE | PPV | TP | FP | FN | SE | PPV | TP | FP | FN | SE | PPV | TP | FP | FN | SE | PPV |
| 100 | 2,273 | 2,272 | 0 | 0 | 100 | 100 | 2,273 | 0 | 0 | 100 | 100 | 2,273 | 0 | 0 | 100 | 100 | 2,273 | 0 | 0 | 100 | 100 |
| 101 | 1,865 | 1,864 | 4 | 1 | 99.95 | 99.79 | 1,862 | 5 | 3 | 99.84 | 99.73 | 1,865 | 1 | 0 | 100 | 99.95 | 1,864 | 5 | 1 | 99.95 | 99.73 |
| 102 | 2,187 | 2,186 | 0 | 0 | 100 | 100 | 2,187 | 0 | 0 | 100 | 100 | 2,187 | 0 | 0 | 100 | 100 | 2,187 | 0 | 0 | 100 | 100 |
| 103 | 2,084 | 2,083 | 0 | 0 | 100 | 100 | 2,084 | 0 | 0 | 100 | 100 | 2,084 | 0 | 0 | 100 | 100 | 2,084 | 0 | 0 | 100 | 100 |
| 104 | 2,229 | 2,226 | 6 | 2 | 99.91 | 99.73 | 2,230 | 1 | 0 | 100 | 99.96 | 2,228 | 8 | 2 | 99.91 | 99.64 | 2,217 | 63 | 12 | 99.46 | 97.24 |
| 105 | 2,572 | 2,567 | 29 | 5 | 99.81 | 98.88 | 2,550 | 67 | 22 | 99.14 | 97.44 | 2,559 | 15 | 13 | 99.49 | 99.42 | 2,566 | 28 | 6 | 99.77 | 98.92 |
| 106 | 2,027 | 2,027 | 1 | 0 | 100 | 99.95 | 2,025 | 5 | 2 | 99.90 | 99.75 | 2,024 | 2 | 3 | 99.85 | 99.90 | 2,010 | 2 | 17 | 99.16 | 99.90 |
| 107 | 2,137 | 2,135 | 0 | 2 | 99.91 | 100 | 2,135 | 0 | 2 | 99.91 | 100 | 2,137 | 0 | 0 | 100 | 100 | 2,136 | 0 | 1 | 99.95 | 100 |
| 108 | 1,763 | 1,739 | 2 | 24 | 98.64 | 99.89 | 1,741 | 199 | 22 | 98.75 | 89.74 | 1,748 | 13 | 15 | 99.15 | 99.26 | 1,735 | 55 | 28 | 98.41 | 96.93 |
| 109 | 2,532 | 2,531 | 0 | 0 | 100 | 100 | 2,531 | 0 | 1 | 99.96 | 100 | 2,532 | 0 | 0 | 100 | 100 | 2,520 | 0 | 12 | 99.53 | 100 |
| 111 | 2,124 | 2,123 | 3 | 1 | 99.95 | 99.86 | 2,124 | 1 | 0 | 100 | 99.95 | 2,123 | 1 | 1 | 99.95 | 99.95 | 2,123 | 3 | 1 | 99.95 | 99.86 |
| 112 | 2,539 | 2,538 | 0 | 0 | 100 | 100 | 2,538 | 0 | 1 | 99.96 | 100 | 2,538 | 2 | 1 | 99.96 | 99.92 | 2,539 | 4 | 0 | 100 | 99.84 |
| 113 | 1,795 | 1,794 | 0 | 0 | 100 | 100 | 1,795 | 0 | 0 | 100 | 100 | 1,795 | 2 | 0 | 100 | 99.89 | 1,794 | 0 | 1 | 99.94 | 100 |
| 114 | 1,879 | 1,879 | 4 | 0 | 100 | 99.79 | 1,862 | 3 | 17 | 99.10 | 99.84 | 1,879 | 3 | 0 | 100 | 99.84 | 1,878 | 2 | 1 | 99.95 | 99.89 |
| 115 | 1,953 | 1,952 | 0 | 0 | 100 | 100 | 1,953 | 0 | 0 | 100 | 100 | 1,953 | 0 | 0 | 100 | 100 | 1,953 | 0 | 0 | 100 | 100 |
| 116 | 2,412 | 2,393 | 1 | 18 | 99.25 | 99.96 | 2,390 | 3 | 22 | 99.09 | 99.87 | 2,411 | 0 | 1 | 99.96 | 100 | 2,392 | 3 | 20 | 99.17 | 99.87 |
| 117 | 1,535 | 1,534 | 0 | 0 | 100 | 100 | 1,534 | 1 | 1 | 99.93 | 99.93 | 1,535 | 1 | 0 | 100 | 99.93 | 1,535 | 0 | 0 | 100 | 100 |
| 118 | 2,278 | 2,278 | 1 | 0 | 100 | 99.96 | 2,275 | 1 | 0 | 100 | 99.96 | 2,275 | 1 | 0 | 100 | 99.96 | 2,278 | 12 | 0 | 100 | 99.48 |
| 119 | 1,987 | 1,987 | 0 | 0 | 100 | 100 | 1,987 | 1 | 0 | 100 | 99.95 | 1,987 | 1 | 0 | 100 | 99.95 | 1,987 | 0 | 0 | 100 | 100 |
| 121 | 1,863 | 1,860 | 0 | 2 | 99.89 | 100 | 1,856 | 4 | 7 | 99.62 | 99.78 | 1,862 | 2 | 1 | 99.95 | 99.89 | 1,862 | 6 | 1 | 99.95 | 99.68 |
| 122 | 2,476 | 2,475 | 0 | 0 | 100 | 100 | 2,475 | 1 | 1 | 99.96 | 99.96 | 2,476 | 0 | 0 | 100 | 100 | 2,476 | 0 | 0 | 100 | 100 |
| 123 | 1,518 | 1,518 | 1 | 0 | 100 | 99.93 | 1,518 | 0 | 0 | 100 | 100 | 1,518 | 0 | 0 | 100 | 100 | 1,518 | 0 | 0 | 100 | 100 |
| 124 | 1,619 | 1,618 | 0 | 0 | 100 | 100 | 1,619 | 0 | 0 | 100 | 100 | 1,619 | 0 | 0 | 100 | 100 | 1,619 | 1 | 0 | 100 | 99.94 |
| 200 | 2,601 | 2,599 | 3 | 1 | 99.96 | 99.88 | 2,598 | 6 | 3 | 99.88 | 99.77 | 2,600 | 0 | 1 | 99.96 | 100 | 2,601 | 47 | 0 | 100 | 98.23 |
| 201 | 1,963 | 1,960 | 0 | 3 | 99.85 | 100 | 1,953 | 0 | 10 | 99.49 | 100 | 1,951 | 1 | 12 | 99.39 | 99.95 | 1,960 | 0 | 3 | 99.85 | 100 |
| 202 | 2,136 | 2,132 | 0 | 3 | 99.86 | 100 | 2,132 | 0 | 4 | 99.81 | 100 | 2,135 | 0 | 1 | 99.95 | 100 | 2,134 | 0 | 2 | 99.91 | 100 |
| 203 | 2,980 | 2,959 | 8 | 21 | 99.30 | 99.73 | 2,952 | 53 | 30 | 98.99 | 98.24 | 2,958 | 2 | 24 | 99.20 | 99.93 | 2,927 | 27 | 53 | 98.22 | 99.09 |
| 205 | 2,656 | 2,654 | 1 | 1 | 99.96 | 99.96 | 2,654 | 0 | 2 | 99.92 | 100 | 2,655 | 0 | 1 | 99.96 | 100 | 2,655 | 0 | 1 | 99.96 | 100 |
| 207 | 1,860 | 1,860 | 5 | 0 | 100 | 99.73 | 1,858 | 4 | 4 | 99.79 | 99.79 | 1,859 | 2 | 3 | 99.84 | 99.89 | 1,853 | 13 | 7 | 99.62 | 99.30 |
| 208 | 2,955 | 2,944 | 3 | 10 | 99.66 | 99.90 | 2,942 | 4 | 14 | 99.53 | 99.86 | 2,952 | 0 | 4 | 99.86 | 100 | 2,925 | 5 | 30 | 98.98 | 99.83 |
| 209 | 3,005 | 3,003 | 2 | 0 | 100 | 99.93 | 3,004 | 3 | 0 | 100 | 99.90 | 3,004 | 0 | 0 | 100 | 100 | 3,005 | 1 | 0 | 100 | 99.97 |
| 210 | 2,650 | 2,646 | 3 | 3 | 99.89 | 99.89 | 2,639 | 2 | 8 | 99.70 | 99.92 | 2,644 | 3 | 3 | 99.89 | 99.89 | 2,621 | 8 | 29 | 98.91 | 99.70 |
| 212 | 2,748 | 2,747 | 0 | 0 | 100 | 100 | 2,748 | 0 | 0 | 100 | 100 | 2,748 | 0 | 0 | 100 | 100 | 2,748 | 1 | 0 | 100 | 99.96 |
| 213 | 3,251 | 3,249 | 0 | 1 | 99.97 | 100 | 3,249 | 1 | 2 | 99.94 | 99.97 | 3,251 | 0 | 0 | 100 | 100 | 3,248 | 2 | 3 | 99.91 | 99.94 |
| 214 | 2,262 | 2,259 | 2 | 1 | 99.96 | 99.91 | 2,258 | 2 | 4 | 99.82 | 99.91 | - | - | - | - | - | 2,259 | 3 | 3 | 99.87 | 99.87 |
| 215 | 3,363 | 3,362 | 1 | 0 | 100 | 99.97 | 3,362 | 0 | 1 | 99.97 | 100 | - | - | - | - | - | 3,363 | 0 | 0 | 100 | 100 |
| 217 | 2,208 | 2,208 | 1 | 0 | 100 | 99.95 | 2,202 | 4 | 6 | 99.73 | 99.82 | 2,207 | 1 | 1 | 99.95 | 99.95 | 2,206 | 1 | 2 | 99.91 | 99.95 |
| 219 | 2,154 | 2,154 | 0 | 0 | 100 | 100 | 2,154 | 0 | 0 | 100 | 100 | 2,154 | 0 | 0 | 100 | 100 | 2,154 | 0 | 0 | 100 | 100 |
| 220 | 2,048 | 2,047 | 0 | 0 | 100 | 100 | 2,048 | 0 | 0 | 100 | 100 | 2,048 | 0 | 0 | 100 | 100 | 2,048 | 0 | 0 | 100 | 100 |
| 221 | 2,427 | 2,427 | 0 | 0 | 100 | 100 | 2,427 | 2 | 0 | 100 | 99.92 | 2,420 | 0 | 7 | 99.71 | 100 | 2,423 | 0 | 4 | 99.84 | 100 |
| 222 | 2,483 | 2,478 | 0 | 4 | 99.84 | 100 | 2,403 | 101 | 81 | 96.74 | 95.97 | 2,475 | 1 | 9 | 99.64 | 99.96 | 2,483 | 3 | 0 | 100 | 99.88 |
| 223 | 2,605 | 2,604 | 0 | 0 | 100 | 100 | 2,605 | 1 | 0 | 100.00 | 99.96 | 2,603 | 0 | 2 | 99.92 | 100 | 2,604 | 0 | 1 | 99.96 | 100.00 |
| 228 | 2,053 | 2,050 | 9 | 3 | 99.85 | 99.56 | 2,048 | 25 | 5 | 99.76 | 98.79 | 2,046 | 3 | 7 | 99.66 | 99.85 | 2,047 | 76 | 6 | 99.71 | 96.42 |
| 230 | 2,256 | 2,256 | 0 | 0 | 100 | 100 | 2,256 | 1 | 0 | 100 | 99.96 | 2,256 | 0 | 0 | 100 | 100 | 2,256 | 2 | 0 | 100 | 99.91 |
| 231 | 1,571 | 1,570 | 1 | 0 | 100 | 99.94 | 1,886 | 0 | 0 | 100 | 100 | 1,886 | 0 | 0 | 100 | 100 | 1,571 | 0 | 0 | 100 | 100 |
| 232 | 1,780 | 1,780 | 6 | 0 | 100 | 99.66 | 1,779 | 6 | 1 | 99.94 | 99.66 | 1,780 | 0 | 0 | 100 | 100 | 1,780 | 20 | 0 | 100 | 98.89 |
| 233 | 3,079 | 3,077 | 0 | 1 | 99.97 | 100 | 3,078 | 0 | 1 | 99.97 | 100 | 3,079 | 0 | 0 | 100 | 100 | 3,072 | 0 | 7 | 99.77 | 100 |
| 234 | 2,753 | 2,753 | 0 | 0 | 100 | 100 | 2,753 | 0 | 0 | 100 | 100 | 2,753 | 0 | 0 | 100 | 100 | 2,752 | 0 | 1 | 99.96 | 100 |
| total | 109,494 | 109,357 | 97 | 107 | 99.90 | 99.91 | 109,532 | 507 | 277 | 99.75 | 99.54 | 104,072 | 65 | 112 | 99.89 | 99.94 | 109,241 | 393 | 253 | 99.77 | 99.64 |
